# “Without this journal, I am in utter darkness”

**Published:** 2018-02-08

**Authors:** Elmien Wolvaardt Ellison

**Affiliations:** 1 Editor: *Community Eye Health Journal,* International Centre for Eye Health, London School of Hygiene & Tropical Medicine, London, UK.

**Thirty years and 100 issues call for celebration and reflection. In this article, the editors and the journal team do both, while the current editor considers the impact the *Community Eye Health Journal* has on its readers and the patients they serve.**

**Figure:**
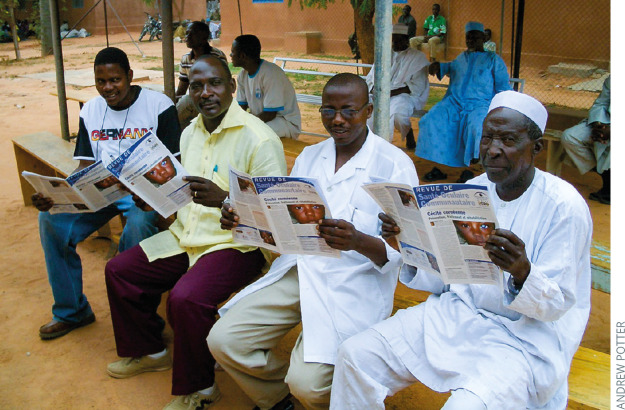
Taking time to read the *Community Eye Health Journal*. NIGER

My interview for the position as editor of the *Community Eye Health Journal* (the *Journal*) is something I will never forget.

The International Centre for Eye Health (ICEH) had moved up in the world – away from the somewhat cramped quarters near Moorfields Hospital from which Dr Murray McGavin published the first issue in 1988, to the lofty heights of the London School of Hygiene and Tropical Medicine's Bedford Square offices in a beautiful three-storey house overlooking a city garden. Perhaps the *Journal* itself hadn't moved quite as far up in the world, however: my interview took place in the basement, headquarters of ICEH's education activities.

Having arrived from South Africa just one month earlier, I had no inkling that I would be spending the next eleven years of my life as editor of this hugely respected journal; working first in that basement and later at the School's Keppel Street building, just around the corner from the house where anaesthesia (chloroform) was first used.

At the interview, Clare Gilbert, Daksha Patel and IAPB CEO Dick Porter subjected me to very thorough but friendly grilling, and I was delighted when the phone call came to say the job was mine: not only would I be working with wonderful colleagues, but I would be contributing to eye health in my own country, even though I now lived so far away.

Of course, I had assumed that my years of experience in journalism, editing and science writing had impressed the panel – only to find out later that it was my enthusiasm for previous editor Victoria Francis's beautiful woodcut panels that had clinched the deal!

**Personal view Dr Murray McGavin**

***Founding editor***

**Figure:**
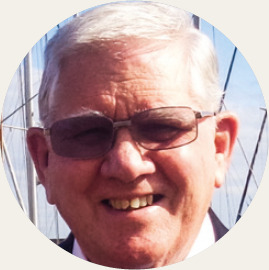

It was while working in Afghanistan that thoughts of an Eye *Journal* to meet the needs of eye care workers in low-income countries first came to me. I recall a ward round in NOOR Eye Institute in Kabul, where patients with severe ocular injuries often came late for eye care. Landmines caused horrific injuries, including blindness. Little did I appreciate then that, 10 years later, Issue 24 would be the first publication worldwide to focus on the blinding effects of landmines.

After returning to Scotland in the mid-1980s, Dr Jock Anderson and Professor Barrie R Jones asked me to join them in the newly established International Centre for Eye Health, then based at Moorfields Eye Hospital in London. I shared my vision of a journal for eye workers in low-income countries with them, and later with Professor Gordon Johnson.

Together with Gordon, we secured funding from CBM, Sightsavers and Coca-Cola in London. CBM and Sightsavers have remained faithful supporters of the *Journal* over the last 30 years and, without them, the Journal would not have been possible. Thank you.

It was my privilege to be the editor of the *Journal* for 15 years, from Issue 1 to 47. When I stepped down, I handed over to the extremely capable health educator Victoria Francis. Our current editor, Elmien Wolvaardt Ellison, joined in 2007 and is continuing this important work.

I am extremely grateful to the many people who joined in the work: the Editorial Boards; the Regional Advisers; colleagues at the International Centre for Eye Health – Professors Gordon Johnson, Allen Foster, Clare Gilbert and Darwin Minassian – who provided advice and opinion. Particular mention is due to Sue Stevens, our Nurse Consultant, whose immense practical contribution and commitment over many years kept us on course as a primary health care publication. I also offer a special word of thanks to Anita Shah, our Editorial Assistant and Administrator. Anita has lasted the pace and coped with different bosses … admirably! Well done, Anita. The emphasis has rightly been on teamwork. So many other names could and should have been mentioned. But, for all involved, Thank you!’

I'm so grateful to have been given the opportunity to start the *Journal* and to see it continuing to support and inform eye care workers around the world. May the *Community Eye Health Journal* continue to fulfil its important function of informing eye care workers in low and middle income settings as they work to prevent and treat blindness worldwide!

## Early days

Just three months later, I was sent back to my home town of Durban for the inaugural World Congress on Refractive Error; my task was to gather ideas and authors for our upcoming issue on that theme. There, for the first time, I discovered how lucky I was to see well despite having high myopia: there are millions of people worldwide cannot not work or read because they do not have a pair of spectacles. A real eye opener. I also started to meet, for the first but not the last time, the many leading lights in international eye health who would be encouraging and supporting me in this work for many years to come, whether by answering questions, writing or reviewing articles, or providing images. I am deeply indebted to all of you.

## A happy challenge

When I started in 2007, the world of international eye health was completely unfamiliar. In fact, to my great embarassment, it was a month or two before I got the better of that pesky extra ‘h’ in ophthalmology! But this did have its advantages: every issue and topic was – and still often is – new to me. I have relished the challenge of learning more about eye health with every issue we produce. For me, the best part is discovering – and then sharing – the ‘why’, and the ‘what needs to change’: why should this issue or theme matter to our readers and their patients? What do we want readers to do about it? And what information do we need to provide to make this possible? In that sense, *the Journal* is an important tool for the improvement of global eye care.

Coming to each theme without specialist knowledge has also helped to ensure that everything we publish is clear to all our readers, many of whom have English as an additional language. It is hard work for everyone involved, and our authors, reviewers and consulting editors have been wonderfully patient with the endless rounds of revisions. We always get there in the end!

## Our readers: my inspiration

What keeps me going is the appreciation I have for every single one of our readers who work, often in the most difficult of circumstances, to prevent blindness and improve the eye health of people in their communities. It is a joy to support them in this work.

Thanks to the excellent readership survey Victoria had conducted in 2005, it was clear to me how much the *Journal* meant to our readers. We repeated her survey, with minor changes, in 2010. I was deeply touched by the lovely things readers said then too. However, it was this comment, from an optometrist in Nigeria, that made the biggest impact. He wrote: *“Without this journal, I am in utter darkness.”* This image has stayed with me ever since, reminding me how much our readers rely on the *Journal* to support them and guide their way.

A few years later, Daniel Etya'ale, another leading light in international eye care, put it into context for me. “When you get to the end of the road somewhere in the middle of Africa,” he said, “and then walk another few hours into the bush, there you will find a clinic with a single ophthalmic nurse who only had her study notes from 20 years ago – until she received *the Journal.”*

**Victoria Francis**

***Editor 2004–2007***

**Figure:**
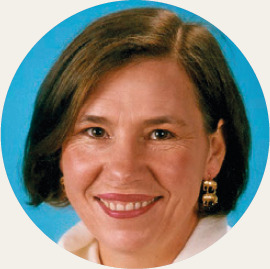

My first issue was SICS – Small Incision Cataract Surgery.

“Why start me off with such a technical topic?”

Allen Foster: “Because if it were too easy you would run away!”

I was the first non-ophthalmologist editor and I remember my gratitude to the editorial committee for their guidance during lively editorial meetings and email exchanges.

Coming from an education, social research and health communication background, I saw my role, at that point in the Journal's life, to review and enhance the way it communicated with its readers. It was important that we were reaching the right people with the right information presented in the clearest way possible. My first task was a content analysis of past issues to review topics and relevance to different levels of eye care workers in diverse geographical settings. We also conducted a reader survey to solicit reader views, particularly how CEHJ influenced attitudes and practice. This, along with dialogue with regional consultants, informed the changes.

The Journal's ‘face lift’ targeted content and design. The Cochrane Eyes and Vision Group contributed a section on “Evidence-based Ophthalmology” and a new “Exchange” section encouraged readers to share inspiring experiences. For the restyle, I worked with talented designer Lance Bellers to maximise space within the 16 pages and modernise layouts leading to the now familiar design starting with issue 51. We tried to make it more visual, using my illustrations as design elements and info-graphics and included ready-to-use education materials, as requested in the reader survey.

Revisiting the 14 issues I worked on, I am reminded of the facilitative relationships with authors – all of them practitioners who made time in very busy lives to share their knowledge and experience. I remember too the fruitful collaborations for the Indian edition and the French translation.

Around this time, the online version was gaining momentum with Sally Parsley delivering improvements in **www.cehjournal.org**. I remember with some shame my scepticism, sticking stubbornly to the argument that many readers would be hard pressed to have a working computer let alone internet access. Comparing Internet World Stats between 2004 and 2017 leads me to eat my hat; Sally's vision was well founded: Africa up from 1% – 31%, Asia up from 7% to 46%, Middle East 7% to 59%, Latin America and the Caribbean 9% up to 61%.

My least favorite aspect of the job was meticulous proofreading to ensure correctness and consistency. To make my life easier, and with a thought for my successor, a small team (kept in check by editorial administrator Anita Shah) made a tedious task fun as we created the Author Guidelines and Style Sheet.

Four years after taking on the editorship, it was time to hand over the baton. My last issue was Research and Training Programmes, a topic close to my heart, and I was delighted to include 20 summaries of Community Eye Health MSc dissertations, demonstrating the close relationship between training offered at the LSHTM and the Journal as a vehicle for providing distance learning to readers across over 180 countries.

Handing over to Elmien and the continuity of the Editorial Committee, I felt confident that the important role of the CEHJ was in safe hands.

**Feedback and questions**

Is there anything you would like us to cover in a future issue? Send questions and suggestions to **admin@cehjournal.org**

## Teamwork for eye health

The *Journal* inspires and enables positive changes in readers' approach to patients and eye disease. We have encouraged readers to communicate better with patients and their families, to actively include women and people with disabilities in their services, and to collaborate with colleagues in other departments to address emerging diseases such as diabetic retinopathy, glaucoma and retinopathy of prematurity. More than anything else, we have promoted the importance of the eye health team: people with different skills and qualifications, working together to improve eye health.

Producing the *Journal* is very much a team effort too. I am very lucky to work with ICEH's inspirational co-directors, Allen Foster and Clare Gilbert; and with the wonderful Nick Astbury (advisor), Anita Shah (editorial administrator), Lance Bellers (designer), and online team Astrid Leck and Miyo Hanazawa. Thank you for everything you do, every day, to ensure that the journal is helpful, beautiful and reaches everyone who needs it.

I would also like to express my heartfelt thanks to our two previous editors, Murray McGavin and Victoria Francis. I am standing on the shoulders of giants.

It is an honour to be here at this important milestone. Here is to another 100 issues!

**Figure:**
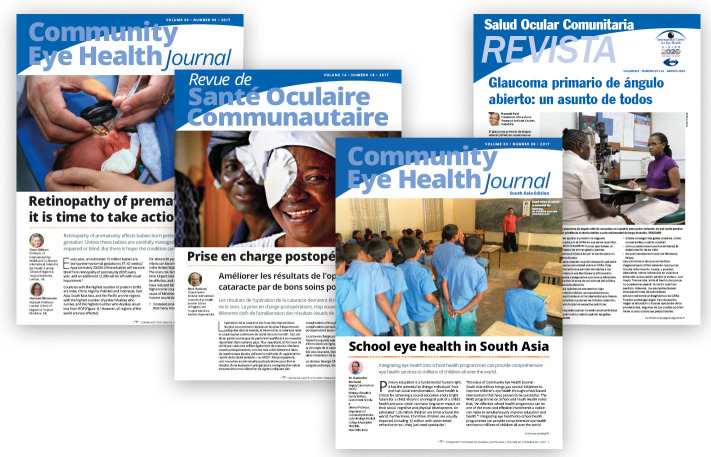
The four editions *(from left to right)*: International, Francophone Africa, South Asia and Latin America.
