# Supplementary material for: “Without this journal, I am in utter darkness”
Source: Community Eye Health. 2018 Feb 8;30(100):102–5. (PMC5820639)
Supplement: Related texts

# Brief report: Reader survey: 2015/2016

**Published:** 2017

**Our readers are at the centre of all our editorial decisions about themes and articles. Readers send feedback throughout the year, and we actively invite readers' input regarding future themes and topics by conducting reader surveys every five years.**

Our most recent survey was conducted by mobile phone. A total of 89 readers from 27 countries were interviewed by then intern Nnamdi Nwuba. Readers were selected to represent the different occupations in eye care: mid-level personnel (ophthalmic nurses, cataract surgeons and ophthalmic clinical officers), ophthalmologists, optometrists and managers/others. They worked in government (57%), the private sector (23%), and non-governmental organisations (17%).

## How useful was *the Journal?*

Three-quarters reported reading either ‘all of’ or ‘most of’ the *Journal*, and nearly two-thirds shared their copy with colleagues. Exactly half of those interviewed used the pictures to explain eye conditions to patients, and 39% relied on the *Journal* when teaching eye health professionals, both formally and informally.

## What did people like about it?

The appreciation for the journal was overwhelming. The following themes emerged very strongly:

- The *Journal* is a source of education and offers an opportunity to connect with the wider world, especially in remote areas
- The information is relevant and useful, particularly in low-income settings
- The *Journal* builds confidence and inspires readers to learn and do more; the international perspective is part of this.

Other comments focused on the format *of* the *Journal*, e.g. the fact that it covers a different theme in-depth in each issue and that it has regular sections such as the editorial article, the quizzes, the announcement section and so on. Readers felt the *Journal* was easy to understand and helpful for teaching others.

**Personal view Anita Shah**

***Editorial administrator***

Dr Murray McGavin, founded the *Community Eye Health Journal* in 1988. I was appointed as his secretary in 1997 and joined Ann Naughton and Sue Stevens to complete the small team responsible for the production of the Journal.

As Editorial Administrator, my overall purpose is to ensure the smooth running of the Journal's production, from planning meetings through to proofreading, printing and distribution. The Journal has a circulation of about 20,000 (International and French CEHJ).

I manage the database and help to increase subscriptions through promotion of the Journal at external events and through correspondence with subscribers. Such direct contact with our readers and authors is a particularly enjoyable part of my role; I am also the first point of contact for many of them.

Coordination of the production schedule involves liaising with editors of other language editions, the designer, the printers, and the website manager. I also manage the article submission and review process of unsolicited research articles and correspond with authors, reviewers and the Editor. All unsolicited articles are sent to two reviewers who are experts in the relevant field of ophthalmology. All comments received are passed back to the authors for the necessary changes to be made. If the article is rejected, feedback is offered with encouragement for future submission, as appropriate.

It is a huge privilege to be part of ICEH, especially the *Community Eye Health Journal* production team and play a part in the promotion of eye health and prevention of blindness worldwide.

*To subscribe, contact Anita on* **admin@cehjournal.org**

## The impact on readers and their patients

Readers were asked: “Has anything you've read or seen in *the Journal* changed the way you work or do your job?” 83% of the respondents said ‘yes’. Change was experienced in many different areas, including clinical and patient care, in readers themselves, and in public health eye care and planning. Here are some examples.

*I used the advice on the management of bacterial conjunctivitis and the management of corneal ulcers. I have since changed my choice of antibiotics. **Cataract surgeon, Zambia***

*It helps practically with going into the community. For example, I wasn't well practised with a Schiotz Tonometer, but I followed step-by-step what you mentioned and it helped me all the way! **Optometrist, Nigeria***

*We now have protocols in place for ophthalmic assistants. It has helped with human resource training. Also, the* Journal *has been useful for the care of ophthalmic instruments. **Ophthalmologist, Kenya***

*We used to focus only on cataracts and glaucoma. Now we can do refractive error and low vision during outreach in the community. We have improved our training programme. We get people from East Africa now, not just West Africa (as we did before). It is a very comprehensive journal! **Eye care coordinator/manager, The Gambia***

*I always wanted to go into the community. It informs me about things to do in the community. Gives me courage to do things. It enables me to assess myself and my ability. I find joy in reading things through and practically performing them. Makes me proud to be an ophthalmic nurse. Even when the doctor is not available, I can feel confident. **Ophthalmic nurse, Nigeria***

*It's had a complete effect on my work. I take ideas from the Journal and use it to revise my work. It directs me to do good things. When I read it, it makes me a better person – better in thinking, better in performing. **Professor/educator, Bangladesh***

*The thoroughness seen in the Journal motivates me to implement changes in my own practice. **Ophthalmologist, Nigeria***

## Internet access

According to the World Bank Development Indicators^1^, internet access in sub-Saharan Africa is still low. Access is highest in Southern Africa (just below 25%) and lowest in Central Africa (just over 10%). The figures were higher among the mobile phone users we interviewed: 44% had access to our online journal and 22% were able to download PDFs to read later (Figure 1). A further 12% had a tablet device, and these higher-earning readers (professors and ophthalmologists) enjoyed being able to share content electronically with others. However, even in this group of readers, 81% said they preferred the paper copy. The majority (61%) reported having problems with internet access, mainly because it was too slow and because access was interrupted as a result of power failures (Figure 2). Congratulations to Sally Parsley, who creating our hugely successful online issue (receiving around 20,000 unique page visits per month!) and to Astrid Leek, who is carrying on this important work. We plan to further develop our online and mobile content, while ensuring that those who need the printed issue receive their copies in the post.

**Figure 1:** How respondents accessed the *Journal* (%). Readers could select any that applied

**Figure 2:** Problems accessing the internet (%)

**Personal view Lance Bellers**

***Graphic designer***

I think I may have said something like “I'll just help a bit on one or two,” to Victoria when we first discussed how best to piece the *Journal* together. That was issue 51 and I'm still here – making it a round 50 issues for me and a perfect 100 for the *Journal* since its inception.

I was working as freelance graphic designer for Sightsavers at the time. When asked if I'd be interested in branching out to work on a project at the International Centre of Eye Health, of course I said yes.

My background is largely reports and magazines – including 20 years on *Focus on Africa* magazine at the BBC World Service – and so my role with *the Journal* is to lend my experience of presenting stories and information in an accessible way to a wide range of readers. The *Journal* is sometimes quite specialist and technical, so it is important that the design makes everything clear and easy to follow. Then everyone can get as much as possible from its pages; whether for their own education, to train others, or to explain eye conditions to patients.

The idea that it is used and appreciated by the readership is very important to me and and it has been a privilege to play a part in its evolution. Elmien has brought her keen journalistic approach to each of her issues since Victoria handed over the baton and it has also been a pleasure for me to design the French edition of the Journal with Paddy Ricard, who works tirelessly to reach the Francophone readership across the world.

In the most recent refresh of both the English and French editions, we have kept in mind the ever-more important online aspect and hopefully the structure lends itself to transferring to the web after each printed edition has come off the press.

The high point? In 2005, I was fortunate to travel in Africa and visit eye care projects in Tanzania and Ethiopia. The look of glee on an elderly woman's face as her bandage was removed after a cataract operation was priceless. The *Journal* helps make this happen – long may it continue.

## References

[1] AgyepongIASewankamboNBinagwahoAColl-SeckAMCorrahTEzehA et al. The path to longer and healthier lives for all Africans by 2030: the Lancet Commission on the future of health in sub-Saharan Africa. Lancet 2017. **http://wdi.worldbank.org/table**10.1016/S0140-6736(17)31509-X28917958
